# A hybrid concept analysis of children of concern: Japanese healthcare professionals’ views of children at a high risk of developmental disability

**DOI:** 10.1186/s12887-016-0715-6

**Published:** 2016-10-28

**Authors:** Ayako Ide-Okochi, Etsuko Tadaka

**Affiliations:** Graduate School of Nursing, School of Medicine, Yokohama City University, 9-3 Fukuura, Kanazawa-ku, 236-0004 Kanagawa, Japan

**Keywords:** Developmental disabilities, Autism spectrum disorder, Child maltreatment, Concept analysis, Infant medical checkup, Screening

## Abstract

**Background:**

The new Diagnostic and Statistical Manual of Mental Disorders (fifth edition, DSM-5) redefined the boundaries of autism as a spectrum. It has been reported that the number of schoolchildren with undiagnosed developmental disorders (DDs) has risen in Japan. Such children referred to as *kininaru-kodomo* (KK, “children of concern”) by healthcare professionals fall into a gray area. Therefore, KK are often overlooked at infant medical checkups. This leaves KK without necessary medical care and special needs education. It is urgent to explore the KK concept to enable professionals to properly assess and provide for the healthcare needs of these children at a high risk of DD, ideally with early intervention.

**Methods:**

A hybrid model of concept analysis was conducted. Working definitions were obtained from a systematic literature review in the theoretical phase. Subsequent in-depth personal interviews initiated in the fieldwork phase corroborated and refined the concept. These qualitative data were integrated in the final analytical phase to yield the practice-based real definition of KK in clinical settings.

**Results:**

Three themes emerged regarding KK children: children who require special care, children whose special healthcare needs are owing to both individual and environmental factors, and children waiting for the development of a new support system for them or their parents.

**Conclusions:**

This study implies that KK are children who require special support because of individual and environmental factors. The concept of KK is considered useful for keeping children with undiagnosed DDs and/or other healthcare needs connected with support networks. It is strongly recommended that a screening tool be developed that reflects the concept of children at a high risk of DD so that children in this gray area may receive necessary support even before diagnosis.

## Background

The fifth edition of the Diagnostic and Statistical Manual of Mental Disorders (DSM-5) introduced new boundaries for autism as a spectrum [1]. The boundaries are symptomatic thresholds dividing the continuum of children with autism spectrum disorders (ASD), children with autistic behaviors, and normal children [2]. In keeping with this continuum theory, it has been proposed that even in the general population, individuals with autistic traits exist, although the severity of their traits is clinically sub-threshold [2, 3]. This extension of the population with social-cognitive difficulties represents the trend of refining the autism diagnosis to reduce the number of people without adequate care despite having social, cognitive, and emotional difficulties [3, 4]. As a result, fewer cases can be diagnosed in childhood, and, needless to say, diagnosis in adulthood is difficult or impossible [4]. In particular, identification of persons with high-functioning autism has long been a problem [4]. In addition to autism, there are other developmental disabilities with underlying neurobiological abnormalities, for example, conduct disorder (CD) [5], attention-deficit/hyperactivity disorder (ADHD), developmental coordination disorder (DCD), and global developmental delay (GDD) [6]. Globally, it is known that children with these developmental difficulties often go undiagnosed and are thus deprived of the opportunity for early intervention [3, 4, 6]. Moreover, the developmental outcome for these children is associated with psychosocial factors such as socioeconomic status and maternal mental health, as well as access to and compliance with treatment [6, 7]. Therefore, in many areas around the globe, it is now recommended to provide early intervention for a wide range of children who are considered at risk of developing behavioral and emotional disorders, to prevent future social exclusion as well as their parents’ difficulties (for example in Australia and Sweden [8, 9]).

The Japanese Ministry of Education, Culture, Sports, Science and Technology announced the results of a national survey in 2012 in which 7.7 % of elementary school students enrolled in a regular class were considered to have developmental disorders (DDs). These children were mostly undiagnosed and more than 90 % were not receiving any special needs education [10]. These children have been referred to as *kininaru-kodomo* (KK, “children of concern”) in Japan. KK have been thought to be children with possible, undiagnosed DDs, and were not considered rare. It has already been reported in Japan that people with undiagnosed DDs exist across age groups [11]. KKs are thought to have trouble adjusting to school life, and maladjustment among schoolchildren has become such a social problem that the government of Japan has suggested an earlier start to compulsory education [12]. Beyond Japan, globally, there is an urgent need to screen children who are thought to be at risk of DDs but who fall into a diagnostic gray area [2, 3]. This study explores the clinical and psychosocial factors present in children at a high risk of DD. For this purpose, we use the concept of KK as a practical example.

The concept of KK has varied over time and across disciplines. KK was first used by middle school teachers to describe truant students with behavior problems [13]. Healthcare professionals may now more generally use this term to mean children with mild DDs. However, the supposed relationship between KK and children with DDs has not been closely examined. For example, it is not obvious that KK would include children who have autistic traits that are under the diagnostic threshold, or children who did not have DDs at the time of infant health checkups. Therefore, it is important to explore the concept of KK to develop a screening system of children with DDs that are either above the threshold or under the threshold in order to provide early support.

Pediatricians and public health nurses (PHNs) have paid attention to this concept [14, 15]. Preschool teachers are also interested stakeholders for these children with undiagnosed and supposed DDs [16]. Previous studies conducted at preschools in an urban setting have reported that 13.3 % of 3-year-old preschoolers were assessed as KK because they possessed autistic traits although they were not diagnosed [17]. In spite of this high awareness of KK, the concept of KK has not been clarified so that each professional can use this term in various ways based on their clinical and educational experiences. In keeping with the mixed definitions, previous studies have shown divergent results regarding age gaps in KK. One study showed that the rate of KK decreased among children from 3 to 5 years old [18]. However, it is more often reported that as preschoolers become older, KK becomes more prevalent [19]. Moreover, assessment differences have been reported when different types of professionals assess the same child’s special healthcare needs [20]. Such discrepancies lead to a failure of children to receive necessary support. Therefore, applying a clear and clinically-useful definition of KK may reduce the frequency of undiagnosed DDs among children and improve treatment.

Professionals on interdisciplinary teams have different explanatory models of the etiology and treatment of language delay [21]. Differences in how each professional defines a disability may affect the distribution of care and affect children’s well-being. In order to improve success in early detection and early intervention, PHNs and day-care-center (DCC) teachers can play an important role because they are the first professionals to meet children with special healthcare needs. PHNs organize infant health check-ups, are trained in screening for children with DDs, and consistently follow up with suspected children [22]. DCC teachers provide daily child care, develop a clear understanding of their children, and are in a great position to detect children’s developmental risks [23]. Each professional is considered part of a collaboration necessary for effective diagnosis and intervention [24]. However, each professional’s concept of KK may differ. This study aims to provide the real meaning of KK by combining the perceptions of professionals in order to promote health and well-being in at-risk children.

## Methods

This study utilized a hybrid model of concept analysis [25] to identify the definition of *kininaru-kodomo* (children of concern) in Japan. This method is considered suitable for exploring the meaning of a concept that has been applied in clinical practice without an explicit definition [25, 26]. For example, withdrawal and pain in elderly patients are somewhat elusive concepts that have been used without universal definitions and explicit measurements [26, 27]. An absence of valid definitions hinders healthcare professionals’ understanding of patients’ conditions. A hybrid model of concept analysis is considered a practice-based approach to allow healthcare professionals’ to understand and measure subjective concepts and enhance the quality of care.

There are three phases: establishing the initial theory, conducting fieldwork, and final analysis. First, to establish the theory, a researcher conducts a systematic literature review to yield a working definition of the concept of interest. The meanings obtained from the existing literature are coded, and these codes are compared to produce categories of aspects of a working definition [25]. Next, the researcher collects and analyzes empirical data to corroborate and refine the working definition based on the practical usage of the concept. By comparing the definitions provided by each participant, categories of aspects of the working definition are also obtained. Finally, the results from the two previous phases are integrated in a final analysis to define the concept and to identify measurement issues and strategies. At this point, the applicability and importance of the concept to related disciplines are also evaluated.

As the first step of concept analysis for KK, we conducted a systematic literature review. We searched Japan Medical Abstracts Society (1983–2013) and Citation Information by National Institute of Informatics (CiNii) Articles (1991–2013). The former search engine includes mainly medical and nursing journals and the latter contains broad areas of articles including education. The search term was “*kininaru-kodomo*.” The search resulted in 349 articles: 100 articles from the former engine and 249 articles from the latter engine. There were few articles prior to the early 1990s but the number of articles gradually increased until it reached almost 40 new articles in 2007, when special needs education in Japan began. We excluded case reports, editorials, and conference repots, and selected 162 articles for full-text review. We included articles on the perceptions of PHNs, DCC teachers, and kindergarten teachers who we considered in this study to be child healthcare professionals. We excluded 128 articles that had no definitions of KK or overlapped with other articles. We added one article regarding a pediatrician’s perception of KK [15] and two articles that studied a related concept of *kininaru-ko* (a child of concern) [16, 17, 28]. *Kininaru-ko* is the singular term of *kininaru-kodomo*, and the latter term is more generally used to refer the troubled children at schools. We conducted our final full-text review of 28 articles.

As the second step, we selected a fieldwork site and conducted semi-structured interviews. The fieldwork phase aimed to obtain practical definitions that were to be compared with the findings of the theoretical phase to refine the working definition. The study site was Yokohama City because public health nurses there were considered to have more advanced knowledge and experiences of the early detection of children with DDs [22]. This city has a population of 3.7 million with 12.9 % juveniles. There are ten rehabilitation centers for children with DDs. Based on the fieldwork methods required for a hybrid concept analysis [27], researchers asked the city bureau to identify model individuals who potentially had a clinical expertise in dealing with KKs. Researchers also intended to include professionals with a wide range of job experiences because knowledge that influenced the person’s definition of the concept might change over time [29]. Six PHNs were recruited from two community health centers located in A and B districts. Both districts were located in the northern, densely populated area of the city. The six PHNs had job experiences of 3 to 25 years. In addition, six teachers from three city DCCs located in A district were recruited. Two DCCs were located in the redevelopment area of the city, in the front of a train station, while the latter one was located in the area where the rate of Okinawan and Brazilian residents was considered relatively high.

The twelve participants were interviewed from December 2013 to January 2014. Participants’ demographic characteristics are presented in Table 1. The interview guide commonly used for both types of professionals included the following items: the meaning of KK, how and when they became aware of KK, how they considered the relationship between KK and children with DDs, and how they collaborate with other professionals when providing KK with support. The interview data were transcribed and analyzed using a grounded-theory approach.

**Table 1:** Demographic characteristics of the participants (*N* = 12)

Characteristics	Number	
	PHNs (*N* = 6)	DCC teachers (*N* = 6)
Gender		
Female	6	5
Male	0	1
Age		
Mean ± SD (range)	36.8 ± 8.6 (24.0–47.0)	37.2 ± 9.4 (28.0–53.0)
Years of current job experience		
Mean ± SD (range)	12.3 ± 7.4 (3.0–25.0)	12.8 ± 10.8 (2.0–29.0)

In the final phase, we integrated the results obtained from the two previous phases to form an overall definition of KK with both theoretical and practical meanings. We paid particular attention to the practical uses of KK in order to make a useful and valid definition that fits with the experiences of professionals. All categories obtained in the previous two phases were compared in terms of relevance to yield themes that seemed justified and supported through literature and fieldwork data [25].

## Results

### The theoretical phase: review of the literature

The literature review revealed three distinct categories for the concept of KK: children with special healthcare needs due to possible DDs, children with special healthcare needs due to other developmental problems, and children with special healthcare needs due to maltreatment (Table 2).

**Table 2:** The working definition of kininaru-kodomo (KK) obtained during the theoretical phase

Working definition	Categories that constitute the working definition	Codes	Relevant references
Children who require special care due to supposed special healthcare needs derived from DDs, other developmental problems and child maltreatment	Children with special healthcare needs due to possible DDs	Children who are assessed to require special care due to possible DDs	32
		Children with undiagnosed DDs that are not accompanied with intellectual delay	15
		Children who have developmental problems including children with diagnosed DDs	16, 17, 18, 19, 28, 31, 32
		Children with problems in behavior and sociability of which professionals are aware	16, 17, 18, 19, 28, 30, 31, 32
		Preschool children with teachers worried about their education	15, 19, 20
	Children with special healthcare needs due to other developmental problems	Children with physical conditions that need medical management	31
		Children with emotional instabilities of professional concern	19, 31
		Children whose life skills are not developed for their age	15, 18
	Children with special healthcare needs due to maltreatment	Children raising professional concern regarding possible child abuse and neglect	30, 31, 32
		Children with mothers with poor child-rearing skills	28, 30, 32
		Children with parents with psychological, emotional, and economic problems	15, 30, 32

The first identified category was children with special healthcare needs due to possible DDs. The reporting of KK as children with possible DDs has increased since 2000. KK has been used as synonymous with children with mild DDs such as learning disabilities (LD), attention-deficit hyperactive disorder (ADHD), and high-functioning pervasive developmental disorder (HFPDD) [10]. The most generally used checklist for KK has been developed based on articles of ADHD and the Child Behavior Checklist [19]. KK has also been used to indicate children with undiagnosed DDs because their developmental problems were not detected at 18- and 36-month infant health checkups [15, 17, 18]. There were conflicting results regarding the relationship between KK and intellectual disability. One leading scholar and pediatrician claimed that KK usually have normal intellectual abilities [15]; however, studies relying on DCC centers reported that teachers also perceived intellectually disabled children as KK [30]. Although KK was not diagnosed, preschool teachers reported awareness of problems with KKs’ behaviors, activities, and sociability, and these teachers felt that KK was associated with troubles with care and education [15, 17, 18]. Preschool children between 2 and 5 years old were perceived as the most typical KK because KKs’ developmental problems become more obvious after group education begins in preschool [15, 18].

The second category identified through literature review was children with special healthcare needs due to other developmental problems. Child healthcare professionals identified children with medical conditions, delayed life skills, and emotional difficulties [31]. Although the relationships between KK and children with DDs prevailed in the literature, professionals were aware of a variety of potential problems that were not always thought to be derived from DDs. Some preschool teachers worried that some KK children may have an allergy [31]. These teachers also reported concerns that KK may refer to children with poor life skills for their age as well as children with apathy [31].

The third category was children with special healthcare needs due to maltreatment by their parents. Children with a variety of problems were included in KK descriptions: child abuse and neglect, inappropriate care at home, parents with mental disability, stress, and living in poverty [23, 31, 32]. With regard to child abuse and neglect, its relationship with KK was first discussed in 2007 in an article on DCC teachers’ perceptions [30]. This category is therefore the most recent to be applied to practice. PHNs provided child-rearing support for mothers of KK, who were assessed to be inexperienced and stressed by child-rearing [32]. DCC teachers admitted that parents of KK children can have problems in their child-rearing abilities such that parents may almost neglect their children or care too intensely [28].

The overall literature review led to the identification of a working definition of KK: children who require special care due to supposed special healthcare needs derived from DDs, other developmental problems, and child maltreatment. PHNs and preschool teachers considered that KK children possessed similar behavioral characteristics with children with diagnosed DDs. All professionals were aware of KKs’ difficulties in studying and playing in a group; however, they also admitted that KK generally had no intellectual delay and that KK were not diagnosed with any disability. Child healthcare professionals perceived KK as children who need special services because of parental maltreatment and/or the parents’ own difficulties.

### Findings of the fieldwork phase

In general, the interview data confirmed the meanings and characteristics specified in the literature. For example, a DCC teacher who had worked at both a DCC and a kindergarten described her perceptions of KK, including all properties of the working definition obtained during the theoretical phase:

“[They are] children whose hyperactivity-like behaviors I have observed. And I think of language development that fails age standards, or a variety of developmental delays. And, well, when telling roughly, through intuition, I immediately know children whose caretakers’ involvement is weak and children’s response is scarce.”

In-depth interviews, however, highlighted some different aspects of the concept. The qualitative analysis identified four new categories for the concept: children with the capacity for positive change, children with ambiguous support needs, and children with a new type of disability, children whose parents require parenting support (see Table 3).

**Table 3:** The meanings of kininaru-kodomo (KK) obtained during the fieldwork phase

Categories	Codes
Children with the capacity for positive change	Children who can catch up with support
	Children whose possible DDs become less severe over time
Children with ambiguous support needs	Children whose behavioral characteristics can be considered as innate or acquired
	Children who fall into a gray area of possible DD
	Children whose needs assessments do not agree between professionals
Children with a new type of disability	Children with a currently increasing type of ASD
	Children who have less severe disabilities than disabled children in the past
Children whose parents require parenting support	Children whose parents have their own medical and social problems
	Children whose parents have never accepted their child’s possible disability
	Children whose parents are supported by intentionally not informing them of possible disability

#### Children with the capacity for positive change

This category was identified in the narratives describing children with possible mild DDs. A DCC teacher with a few decades of job experience admitted that KK had uneven developmental conditions but that they could catch up when their deficiencies were supplemented through support. A PHN in her 30s said that she did not want to label KK children as disabled:

“The characteristics that KK have currently may become less severe with age. In addition, the conditions may come to a better direction. Even if KK have troubles right now, they may be able to get through years later.”

#### Children with ambiguous support needs

There were three types of ambiguities that could lead a child to be classified as KK: individual traits, environmental factors, and diagnostic standards, and all were intertwined. First, professionals considered that assessing the cause of children’s troubles was not an easy task because KK often have both developmental and environmental problems that were intertwined:

“I cannot discern them well. As for the environmental factors, maltreated children have the developmental-disability-like traits. In another case whom I introduced to a rehabilitation center because of the same behavioral characteristics of children with DDs, the center referred that child back because they decided on environmental causes.”

Second, it was considered difficult to discern KK and children with DDs. Although these groups of children often shared similar traits, KK children were not those children that were officially diagnosed. The absence of a diagnosis made DCC teachers especially uneasy, because the diagnosis is a prerequisite to starting special-needs education. In addition, professionals admitted that there was a gray area between KK and children with diagnosed DDs:

“It is very difficult to distinguish KK from children with DDs. I am also concerned about linking those children to children with disabilities. Well, it is difficult to say; the gray zone is really wide.”

Third, professionals indicated that KKs’ problems were so ambiguous that multidisciplinary professionals and parents often cannot share the results of their assessments of a child’s special needs. For example, it was stated that even when DCC teachers were worried about a child’s development, some KK children could easily pass 3-year-old health checkups at the community centers held by PHNs. Therefore, PHNs were making a different conclusion regarding the child’s need for special care.

“At the 36-month health checkup, PHNs observe such children (those whom DCC teachers have indicated to PHNs as of concern) only that day, on one occasion. Well, there are so many children that PHNs cannot sense their developmental problems. As such, I feel a gap between us.”

#### Children with a new type of disability

PHNs said that children with ASD have currently increased in prevalence and that they might overlook autistic children who have high enough functioning that they are able to pass developmental tests conducted at 3-year-old health checkups. Moreover, a DCC teacher with nearly 30 years of job experience indicated her impression of KK children as having a new type disability. This teacher said that she has observed a transition in the type of disabilities children possess when they are admitted to DCCs. She recalled that in the beginning of her career, she cared for children with Down syndrome, and then she saw more children with more severe autism. She considered that KK was the newest wave of child disabilities that are being care for by DCC teachers.

“When such (severe) children went to facilities other than DCCs, now, this time, it is strange to say that these children missed those facilities’ application thresholds, and, well, I feel badly to say, ‘sub-threshold’ children are increasing [in DCCs], who are not so bad in their development but are poor in their behaviors.”

#### Children whose parents require parenting support

Parents of KK were assessed to require parenting support due to their own problems that included parenting behaviors such as yelling at their children, forcing their children to obey, and heavily indulging or doting on their children. Parents’ psychiatric disorders were also considered problems that lead to a need for additional parenting support. A PHN with nearly 15 years of job experience spoke of her experience supporting KK:

“For a case of children with whom I am involved for a long period, I suppose, their parents have their own problems. Therefore, many of them need child-rearing support. In addition, children of those parents have DDs; therefore, they have difficulties in child-rearing.”

In relation to this category, PHNs considered that one potential means of support may be to intentionally not tell parents of their child’s possible disability, until parents can recognize their child’s possible DD on their own:

“For a parent who has never thought of children’s developmental problems, I don’t dare to tell them of their child’s problems.”

### Findings of the analytic phase: conceptual delineation by integrating theory and fieldwork

In the final analytic phase, the findings of both the theoretical and fieldwork phases were compared to yield an integrated and practical definition. The theoretical phase identified three categories of the causes of KK children’s support needs. According to our literature review, more researchers considered KK to be children with possible DDs. Identified difficulties referred to troubles diagnosing possible but undetected DDs and children’s actual troubles in their social life. KK was a widely applied concept that included both children with other developmental problems and possibly maltreated children. On the other hand, the fieldwork phase added new meanings to the working definition that were employed in practice. Although the working definition was essentially confirmed in this phase, interviews with healthcare professionals added new categories regarding beliefs in KK children’s capacity for improvement, the ambiguity of KK children’s problems and related difficulties in needs assessments, the newness of KK associated “disabilities,” and professional’s support styles toward parents of KK children. In particular, professionals indicated that they were perplexed with the ambiguous differences between children with and without diagnoses. In addition, gaps and discrepancies were identified between professionals’ needs assessments of KK children. Professionals were very concerned regarding the increase of children with possible ASD who were thought to have a new type of disability that was not yet being reliably identified and diagnosed by existing standards of health checkups and service eligibilities.

By integrating the literature review and the practical fieldwork results, three themes were identified that embodied the attributes of KK (see Table 4). Hence, the working definition in the theoretical phase was revised to reflect the findings of the fieldwork phase. The final definition obtained (Table 4) was that KK represents children who require special care due to both individual and environmental conditions, but can grow up healthy by applying appropriate support systems. This definition includes the three categories of attributes that were obtained at both of the two preceding phases.

**Table 4:** The final definition of KK through integration of literature data and fieldwork results

The final definition	Themes that constitute the final definition	Categories obtained at the preceeding two phases
Children who require special care due to both individual and environmental conditions, but can grow up healthy by applying appropriate support systems	Children who require special care	Children with special healthcare needs due to potential DDs
		Children with special healthcare needs due to other developmental problems
		Children with special healthcare needs due to maltreatment
		Children with the capacity for positive change
	Children whose special healthcare needs are owing to both individual and environmental factors	Children with special healthcare needs due to potential DDs
		Children with special healthcare needs due to other developmental problems
		Children with special healthcare needs due to maltreatment
		Children with ambiguous support needs
	Children waiting for the development of a new support system for them or their parents	Children with special healthcare needs due to maltreatment
		Children with ambiguous support needs
		Children with a new type of disability
		Children whose parents require parenting support

## Discussion

The aim of this study was to examine the concept of a child’s being at risk of developmental disability by assessing Japanese child healthcare professionals’ views, which reflect clinical and psychosocial concerns. In this way, the current study provided empirical results that could help redraw the boundaries regarding children at risk of developmental disabilities or difficulties [1–3]. This study is the first to explore the definition of *kininaru-kodomo* through a hybrid model of concept analysis that integrates the findings of a literature review with fieldwork interviews. Both theoretical and fieldwork phases suggested a relationship between KK and children with diagnosed DDs. In particular, the fieldwork findings illuminated the professionals’ perceptions of a broad gray area of ASD diagnosis without an obvious, natural cutoff point [2]. The category, “children with ambiguous support needs,” suggested that health professionals were not confident with screening KKs probably because their problems were not severe enough to meet criteria for developmental abnormalities. This study’s findings support previous reports of the phenomenon of children who are absent diagnosis-level autistic traits but who in reality present social-cognitive difficulties [2, 3]. More research is essential to reconsider the boundaries between affected and unaffected children based on a supposed cut-off point within the diagnosis criteria.

In addition to this ambiguity, the difficulties in early screening were considered to be derived partly from the insufficiency of the current child health-checkup system that does little to detect ambiguous developmental problems (although it was thought to be better at detecting developmental delay) [15]. Although early-intervention systems have been introduced that follow up with at-risk children including KK [32], it is more common that children without a diagnosis are not provided necessary support [17, 20]. It has been suggested that services for children with DDs are not well developed in Japan [11]. The scarcity and/or poor quality of services are considered one reason for the maladjustment in adulthood of children with diagnosed autism [33]. Even children with diagnosed DDs have troubles being provided necessary support, and this situation must be even worse for those children without a diagnosis. As it is dependent on each professional’s opinion whether KK children are assessed as at-risk [20], the chance for KK children to receive early support is considered skewed. In Japan, KK children’s maladjustment during transitional periods is already a national concern [34]. In addition to diagnostic features, poor psychosocial functioning predicts future social and economic exclusion in children with mental health problems [35]. The professionals’ narratives of KK could be considered convincing examples of such poor psychosocial functioning, and hence this study could provide the variables relevant to children in need of early intervention.

This study highlights the existence of a gray area of diagnosis of DDs and describes the varieties of children that fall into this gray area through both health and educational professionals’ narratives. Future research should illuminate the gray area between KK and children with ASD, so that more children at potential risk will be included as targets of care. This approach is consistent with the DSM-5′s new concept of ASD as spectrum [1], and this may change the way that professionals consider the boundaries of KK, children with autistic-like traits [2], and children with ASD in their everyday practices.

In addition to further exploration of the concept of KK, it is necessary to develop new child health-checkup systems. In our analysis, we identified a theme of “children who are waiting for the development of a new support system for them and their parents.” Both literature [15] and interview data suggested that professionals perceived their inability to completely detect and diagnose children with DDs using existing child health-checkup systems. The consultation rate of these checkups is relatively high in Japan [22]. However, these checkups will fail to promote early detection of DDs unless they include the assessment of developmental imbalances in addition to developmental delays [15]. Indeed, detection varies across regions, and the follow-up rate after 18-month health checkups ranged from 1.9 to 56.3 % across municipalities in Japan [36]. This study implied that more opportunities should be provided for a child and parent to confirm the possibility of developmental problems via cooperative relationships with health professionals in the community [9].

Moreover, this study implied that children with high-functioning DDs are a relatively new phenomenon in Japanese welfare settings. Therefore, the detection system in place to identify such children “with ambiguous problems” is poorly developed. Thus, it is urgent to develop a new health-checkup system to effectively identify children with possible DDs who might be sub-threshold on current screening tools [2]. Not only in Japan but globally, identification of high functioning autism in the absence of intellectual delay is a persistent problem [4]. In the guideline, it is recommended to use the Autism Spectrum Quotient (AQ) [4]. However, a previous study has shown that even among the general population who fall below the cut-off point for ASD traits as measured by the AQ, there are individuals poorer at social cognition and cognitive-emotional flexibility [3]. Our findings are therefore in line with past research that suggests the need to reconsider current diagnostic systems based on the assumption of a dichotomy between affected and unaffected individuals [2].

One previous review suggested that parenting concerns are closely related to outcomes and that the concerns of parents and teachers are likely to be an effective first screening tool for children at developmental risk, if stigma can be reduced [7]. This study further highlights the need to develop a new screening system that utilizes the perceptions of parents and multidisciplinary professionals who traditionally have not played a major role in mass screening systems of children with any healthcare needs in Japan.

There are several limitations to this study. Interview participants were limited both in number and discipline. Pediatricians and dentists in Japan are medical professionals that also participate in 18- and 36-month health checkups [36]. Therefore, future research should explore their perceptions of KK because they also play an important role in the detection of children’s developmental problems. In addition, the fieldwork was conducted only in one city. This evidence is insufficient to conclude that this concept or the problems identified are present across either the nation or the world. Future nationwide research is necessary to assess the extent of the diagnostic gray area between children with diagnosed ASD and subthreshold children.

## Conclusions

Our hybrid model of concept analysis revealed a clear and practical definition of KK that related to the broad boundaries of the ASD concept. Participating professionals were troubled with their potential inability to detecting, diagnose, and provide proper services for ASD among KK children. These children are often provided scarce support because of discrepancies in professionals’ needs assessments. Another aspect of KK included maltreated children, adding to difficulties in early screening of ASD and ASD-like-trait children because in the view of our participants, maltreatment and DDs were intertwined. Future study is necessary to construct a new system of assessing children’s healthcare needs for those that are sub-threshold on existing standards of screening and detecting DDs.

## Abbreviations

ADHD: Attention-deficit hyperactivity disorder
AQ: Autism spectrum quotient
ASD: Autism spectrum disorders
CD: Conduct disorder
CiNii: Citation information by National Institute of Informatics
DCC: Day-care-center
DCD: Developmental coordination disorder
DDs: Developmental disorders
DSM-5: The fifth edition of the diagnostic and statistical manual of mental disorders
GDD: Global developmental delay
HFPDD: High-functioning pervasive developmental disorder
KK: *Kininaru-kodomo*, “children of concern”
LD: Learning disabilities
PHNs: Public health nurses
